# Doctor, What Shall I Eat?

**Published:** 1882-11

**Authors:** 


### BOOK NOTICES, REVIEWS, ETC.

"Doctor, What Shall I Eat?" A hand-book of diet in disease, for the profession and the people. By Charles Gatchell, M. D. Second edition. Duncan Brothers: Chicago. 1882.

We can recommend Dr. Gatchell's little book to all who need a brief, clear and reliable guide to the dietetics of the sick. It gives no scientific discussions on the principles of feeding, but simply tells one how to feed the sick Useful alike to professional and lay readers.

